# Neuronavigation-guided precise resection versus conventional glioma surgery: a meta-analysis of clinical outcomes

**DOI:** 10.3389/fneur.2026.1893712

**Published:** 2026-09-15

**Authors:** Shuanglei Guo, Kang He, Bingjian Yuan, Xiaobing Chen, Honglin Liu, Zhenjiang Li

**Affiliations:** Department of Neurosurgery, Huaihe Hospital of Henan University, Kaifeng, China

**Keywords:** advanced neuroimaging techniques, brain tumor surgery, extent of resection, glioma surgery, intraoperative MRI, multimodal imaging, neuronavigation, surgical outcomes

## Abstract

**Background:**

Neuronavigational surgery has become a cutting-edge technique that improves surgical accuracy, increases tumor excision rates, and preserves neurological function in glioma patients.

**Aim:**

This meta-analysis was conducted to assess the efficacy of neuronavigational and advanced imaging-assisted surgery compared to traditional glioma surgery.

**Methods:**

In total, 30 articles published between 2006 and 2026, including randomized controlled trials, prospective cohort studies, retrospective cohort studies, and case series, were evaluated. The intervention involved intraoperative MRI (iMRI), ultrasound-guided navigation, functional neuronavigation, and other advanced navigational technologies. Meta-analysis was carried out using random effects models with inverse variance to calculate pooled odds ratios (ORs), 95% confidence intervals (CIs), *I*^2^ and *τ*^2^ measures of heterogeneity, and publication bias using funnel plots and Egger’s regression test. The risk of bias was assessed through the Cochrane RoB 2 for 7 randomized controlled trials and ROBINS-I for 23 observational studies.

**Results:**

Overall analysis demonstrated significant improvement with neuronavigation-guided surgery (OR = 2.37, 95% CI: 2.05–2.74, *p* < 0.05; *I*^2^ = 22.8%). Subgroup analyses showed significant benefits for iMRI-guided surgery (OR = 2.49, 95% CI: 1.92–3.23, *z* = 6.88, *I*^2^ = 22.6%, *τ*^2^ = 0.0428, *p* = 0.2286), ultrasound-based navigation (OR = 1.97, 95% CI: 1.47–2.66, *z* = 4.50, *I*^2^ = 4.9%, *τ*^2^ = 0.0084, *p* = 0.3685), functional neuronavigation (OR = 2.11, 95% CI: 1.59–2.80, *z* = 5.17, *I*^2^ = 0%), advanced navigation technologies (OR = 2.33, 95% CI: 1.67–3.25, *z* = 4.99, *I*^2^ = 16.4%), and other specialized techniques (OR = 2.16, 95% CI: 1.63–2.86, *z* = 5.34, *I*^2^ = 8.3%). Leave-one-out analysis confirmed the stability of the pooled effect, with minimal changes after exclusion of individual studies.

**Conclusion:**

Neuronavigation-based and image-enhanced surgical procedures can significantly improve glioma surgery outcomes, with low heterogeneity and strong pooled effects. Large-scale multicenter studies are needed in order to prove their efficacy in the long term.

## Introduction

Gliomas are among the most frequent primary malignant brain tumors, characterized by a considerable degree of disability and mortality rate. Although substantial progress in the field of neuro-oncology has been achieved, gliomas’ invasive behavior, biological heterogeneity, and proximity to eloquent brain areas pose considerable difficulties for their complete surgical removal (1). Surgery is the gold standard for the treatment of low- and high-grade gliomas, owing to its impact on progression-free survival, overall survival, and quality of life. Nevertheless, traditional microsurgical procedures have a limited ability to distinguish normal brain parenchyma from tumor cells (2–4).

In the last few decades, many technological innovations have been developed to increase the accuracy and safety of glioma operations. One such innovation is the neuronavigation system, which facilitates the integration of images obtained from preoperative imaging modalities into the intraoperative procedure (5). The use of the system increases the accuracy and safety of the operation; however, it may not perform well due to brain shifting that can occur during the process. This has led to the development of intraoperative imaging modalities, including iMRI and iUS (5–7).

Intraoperative MRI has especially become very powerful in today’s neurosurgery. This technology enables surgeons to assess the completeness of resection during the operation and decide on further resection based on their observations (8–10). Several field strengths have been developed for iMRI technology, among which high-field-strength iMRI is more accurate. Likewise, multimodal neuronavigation combines techniques such as fMRI, DTI, and tractography to protect white matter tracts (11–13).

Along with imaging innovations, there have been growing efforts to optimize the surgical strategy. Supratotal resection, awake craniotomy, and fluorescence-based surgery, which uses chemicals such as 5-ALA, represent some of the measures that have been taken to remove tumors with minimal impairment to the patient’s neurological functions. This is indicative of a shift from an anatomy-based procedure to one guided by function. The new techniques being developed include hyperspectral imaging, augmented reality (AR), and biological stratification (14–16).

Because of rapid advancements in neurosurgical techniques and the growing body of evidence demonstrating their effectiveness, it is crucial to conduct an extensive analysis of the existing literature. The analysis of the effectiveness of neuronavigation-guided and imaging-assisted glioma surgery could reveal which method is better and identify gaps in current research (17–20). The present meta-analysis aims to compare neuronavigation-guided and imaging-assisted glioma surgery with traditional methods, considering the extent of resection and the effectiveness of both approaches.

## Methods

### Study design

This study was conducted as a systematic review and meta-analysis following the PRISMA 2020 guidelines, synthesizing randomized controlled trials, prospective and retrospective cohort studies, and case series comparing neuronavigation-guided or advanced image-assisted glioma surgery with conventional surgery, published between 2006 and 2026.

### Eligibility criteria

Studies were eligible for inclusion if they compared neuronavigation-guided or advanced image-assisted surgical resection against conventional (non-navigated or standard neuronavigation) glioma surgery. Eligible populations included adult and pediatric patients with histologically or radiologically confirmed glioma of any grade, including low-grade glioma, high-grade glioma, glioblastoma, diffuse glioma, and brainstem glioma. Eligible study designs comprised randomized controlled trials (RCTs), prospective cohort studies, retrospective cohort studies, and case series reporting extractable comparative or single-arm outcome data. Studies were required to report at least one outcome of interest, as defined in the Data Items section below. Only full-text, peer-reviewed, open-access articles published in English between January 2006 and 2026 were eligible.

Studies were excluded if they were conference abstracts, editorials, narrative reviews, single case reports without extractable group data, studies of non-glioma intracranial pathology, or duplicate/overlapping patient cohorts, in which case the study with the largest or most complete dataset was retained.

Given the molecularly integrated diagnostic framework introduced by the WHO CNS5 classification (2021), molecular biomarker status (e.g., IDH mutation, 1p/19q co-deletion, MGMT promoter methylation) was recorded where reported by included studies. However, molecular subtype was not used as a formal inclusion/exclusion criterion or synthesis-grouping variable, as the majority of eligible studies classified tumors by histological grade alone; a molecular-subtype-stratified subgroup analysis was therefore not performed.

### Comparator definition

The comparator (“conventional surgery”) was defined as glioma resection performed using standard preoperative imaging and/or standard frameless neuronavigation without the specific advanced technology under investigation—for example, microsurgical resection under white-light visualization, or standard neuronavigation without intraoperative imaging updates, functional data integration, or fluorescence guidance—consistent with each study’s own description of its control or reference arm.

### Grouping of studies for synthesis

For the purposes of synthesis, included studies were grouped into five pre-specified, mutually exclusive intervention categories, defined as follows, in addition to an overall pooled analysis combining all 30 included studies:

- Intraoperative MRI (iMRI)-Guided Surgery—resection incorporating real-time or interval magnetic resonance imaging performed within the operating theatre (low-field, e.g., 0.15 T; high-field, e.g., 1.5 T or 3.0 T) to detect residual tumor and guide further resection during the same operative session, used alone or combined with standard neuronavigation.
- Ultrasound-Based Navigation—resection guided by intraoperative ultrasound imaging, including B-mode ultrasound, two- or three-dimensional intraoperative ultrasound (IOUS), and ultrasound combined with neuronavigation registration, used to localize tumor margins and assess extent of resection in real time.
- Functional Neuronavigation—navigation incorporating functional or tractographic data to preserve eloquent brain structures, including diffusion tensor imaging (DTI)-based tractography, functional MRI (fMRI)-integrated navigation, awake craniotomy with cortical/subcortical direct electrical stimulation (DES) mapping, and magnetoencephalography (MEG)-guided navigation.
- Advanced Navigation Technologies—emerging or hybrid image-guidance platforms, including mixed reality (MR)-guided navigation, augmented reality (AR) head-mounted holographic navigation, multimodal navigation protocols combining two or more imaging modalities (e.g., neuronavigation plus intraoperative ultrasound plus iMRI), and standardized multimodality imaging protocols (MRI/MRS/fiber tracking/3D reconstruction).
- Other Specialized Techniques—adjunctive intraoperative visualization or neuromodulation methods not classified above, including 5-aminolevulinic acid (5-ALA) fluorescence-guided resection (standard and delayed-administration protocols), sodium fluorescein-guided resection, peri-operative repetitive transcranial magnetic stimulation (rTMS), supratotal versus gross total resection comparisons under iMRI guidance, and neuronavigation-guided resection specific to brainstem glioma.

Studies were assigned to the category corresponding to their principal navigational technology; where a study compared more than one advanced technique against conventional surgery within the same cohort, it was classified according to its stated primary intervention arm, and this decision was cross-checked against each study’s own description of its methodology.

### Information sources

Electronic databases searched included PubMed/MEDLINE, Embase, Scopus, Cochrane Central Register of Controlled Trials (CENTRAL), and Web of Science, each searched from inception through [insert final search date]. The trial registries ClinicalTrials.gov and the WHO International Clinical Trials Registry Platform (ICTRP) were searched to identify ongoing or unpublished trials (e.g., FUTURE-GB, GLIMMER/SPECTRE). Reference lists of included studies and relevant prior systematic reviews and meta-analyses were hand-searched to identify additional eligible studies, and Google Scholar was used as a supplementary source to capture gray literature. [Author note: replace bracketed placeholders with the verified database list and exact search dates used by the review team.]

### Search strategy

A representative search strategy combined controlled vocabulary (MeSH/Emtree terms) and free-text keywords across four concept blocks joined with Boolean operators, for example:

*(“glioma” OR “glioblastoma” OR “astrocytoma” OR “brainstem glioma”) AND (“neuronavigation” OR “intraoperative MRI” OR “iMRI” OR “intraoperative ultrasound” OR “functional neuronavigation” OR “5-aminolevulinic acid” OR “5-ALA” OR “diffusion tensor imaging” OR “augmented reality” OR “mixed reality” OR “awake craniotomy”) AND (“resection” OR “surgery” OR “surgical outcome”) AND (“randomized controlled trial” OR “cohort study” OR “case series” OR “comparative study”).*

Searches were limited to human studies published in English between 2006 and 2026; no other filters were applied. The complete, verbatim search strategy for each database, including all field tags and limits, should be reported in a Supplementary appendix.

### Selection process

Titles and abstracts of all retrieved records were screened independently by two reviewers against the eligibility criteria, with disagreements resolved by discussion or adjudication by a third reviewer. Full-text articles of records passing initial screening were then independently assessed for eligibility by the same two reviewers, with reasons for exclusion recorded at this stage. Screening was managed using reference-management/systematic-review software, with duplicate-detection tools used to remove duplicate citations prior to screening; no automated machine-learning classification was used to exclude records.

### Data collection process

Data were extracted independently by two reviewers using a piloted, standardized data-extraction form, with discrepancies resolved by consensus or third-reviewer arbitration. Where reported outcome data were incomplete or ambiguous, corresponding authors were contacted for clarification or additional data; if no response was received within 4 weeks, the study was analyzed using only the data available in the published report. No automated data-extraction tools were used.

### Data items

#### Outcomes

The primary outcome was a composite measure of surgical efficacy, defined as a binary (event/no-event) indicator of favorable surgical outcome—most commonly achievement of gross total resection (GTR), complete or near-complete tumor resection on postoperative imaging, or another study-defined threshold of favorable resection or functional outcome—compared between the navigation-assisted and conventional surgery arms. This composite definition permitted pooling of odds ratios across studies with heterogeneous but conceptually aligned primary outcomes.

Secondary outcomes and their operational definitions were as follows:

- Extent of resection (EOR): the percentage of tumor volume removed, calculated as (preoperative tumor volume − postoperative residual tumor volume)/preoperative tumor volume × 100, based on volumetric analysis of pre- and postoperative or intraoperative MRI.
- Gross total resection (GTR) rate: the proportion of patients in each arm achieving complete macroscopic/radiological resection of contrast-enhancing (or FLAIR-defined, for non-enhancing) tumor, as determined by post-resection imaging.
- Residual tumor volume: the absolute or relative volume of tumor remaining on early postoperative or intraoperative imaging following resection.
- Progression-free survival (PFS): the time from surgery (or randomization) to radiological or clinical tumor progression, or death from any cause, whichever occurred first.
- Overall survival (OS): the time from surgery (or randomization) to death from any cause.
- Postoperative neurological/functional deficit: new or worsened neurological impairment (motor, language, or other domain-specific deficit) following surgery, assessed clinically or via standardized scales such as the National Institutes of Health Stroke Scale (NIHSS) or modified Rankin Scale (mRS).
- Karnofsky Performance Status (KPS): a standardized 0–100 scale assessing functional performance and independence, used as a measure of postoperative functional status and quality of life.
- Seizure outcome (where applicable): postoperative seizure control classified using the Engel classification system, extracted for studies involving seizure-presenting low-grade glioma populations.

Where a study reported an outcome under a different label but a conceptually consistent definition (e.g., “complete resection” versus “gross total resection”), it was mapped to the corresponding standardized endpoint above. For all outcome domains, results across all reported measures, time points, and analyses compatible with the domain definition were sought; where multiple time points were available, the longest available follow-up was prioritized for survival outcomes, and the earliest postoperative/intraoperative MRI-assessed value was used for resection-based outcomes. Outcomes not conforming to any of the above definitions, or reported in a form not convertible to odds ratios (e.g., continuous volumetric data without variance estimates), were described narratively rather than pooled quantitatively.

#### Other variables

Additional extracted variables included author, publication year, country, study design, sample size (total and per arm), population and tumor characteristics (glioma grade, anatomical location), specific intervention/technology used, comparator definition, study duration/enrollment period, and funding source where reported. Where sample size or arm allocation was ambiguous, such as in interim analyses of ongoing trials, the number of patients analyzed at the time of publication was used, and this was noted as a limitation. No further assumptions were made about missing or unclear information beyond those stated above.

#### Study risk of bias assessment

Risk of bias in the seven included randomized controlled trials was assessed using the Cochrane Risk of Bias 2 (RoB 2) tool across five domains: randomization process, deviations from intended interventions, missing outcome data, measurement of the outcome, and selection of the reported result. Risk of bias in the 23 included observational studies (prospective and retrospective cohorts, case series) was assessed using the Risk Of Bias In Non-randomized Studies of Interventions (ROBINS-I) tool across seven domains: confounding, selection of participants, classification of interventions, deviations from intended interventions, missing data, measurement of outcomes, and selection of the reported result. Assessments were conducted independently by two reviewers, with disagreements resolved through discussion or third-party adjudication; no automation tools were used in this process.

#### Effect measures

The odds ratio (OR) with 95% confidence intervals (CIs) was used as the effect measure for all outcomes, reflecting the predominantly binary/categorical nature of the reported surgical endpoints (e.g., gross total resection achieved versus not achieved, neurological deficit present versus absent).

## Synthesis methods

### Eligibility of studies for each synthesis

Study intervention characteristics were tabulated (author, intervention technology, comparator, and outcome endpoint) and cross-checked against the five pre-specified synthesis groups described under Eligibility Criteria to confirm appropriate classification prior to pooling.

### Data preparation

Where studies reported raw event counts, odds ratios were calculated directly; where only proportions, means, or hazard ratios were reported, standard conversion formulas were applied to derive comparable log-odds estimates and standard errors. Missing standard deviations or variance estimates were imputed using methods consistent with the Cochrane Handbook for Systematic Reviews of Interventions (e.g., derivation from confidence intervals, *p*-values, or comparable studies) where necessary.

### Tabulation and visual display

Individual study and pooled results were displayed using forest plots for each intervention subgroup and the overall analysis, funnel plots for assessment of small-study effects, and summary tables reporting pooled OR, 95% CI, *z*-value, *I*^2^, *τ*^2^, and Egger’s regression statistics for each synthesis.

### Synthesis model, heterogeneity, and software

A random-effects model using the inverse-variance method was used for all pooled analyses, reflecting the anticipated clinical and methodological heterogeneity across study designs, glioma grades, and navigational technologies. Statistical heterogeneity was quantified using the *I*^2^ statistic (categorized as low [<25%], moderate [25–75%], or high [>75%]) and the *τ*^2^ estimate of between-study variance. R (meta for package) or Review Manager (RevMan) software were used.

### Exploration of heterogeneity

Subgroup analyses were pre-specified by intervention/technology category—iMRI, ultrasound-based navigation, functional neuronavigation, advanced navigation technologies, and other specialized techniques—to explore heterogeneity attributable to navigational modality. Meta-regression was not performed given the limited number of studies within most subgroups.

### Sensitivity analysis

A leave-one-out sensitivity analysis was conducted for the overall pooled estimate, sequentially omitting each of the 30 included studies and recalculating the pooled OR, 95% CI, and *I*^2^ to assess the robustness and stability of the overall effect.

### Reporting bias assessment

Risk of bias due to missing results (publication/reporting bias) was assessed for the overall analysis and for each intervention subgroup using visual inspection of funnel plot symmetry and formally tested using Egger’s regression test, with the intercept, 95% CI, *t*-value, and *p*-value reported for each synthesis. A trim-and-fill analysis was additionally performed to estimate the potential impact of missing studies on the pooled effect estimate.

### Certainty assessment

Certainty in the body of evidence for each outcome was informed by the Grading of Recommendations, Assessment, Development and Evaluations (GRADE) framework, considering risk of bias (from RoB 2/ROBINS-I ratings), inconsistency (*I*^2^/*τ*^2^), indirectness (variation in navigational technology and glioma subtype across studies), imprecision (width of pooled 95% CIs), and publication bias (Egger’s test/funnel plot asymmetry) for each of the five synthesis groups and the overall analysis.

## Results

### Study selection

In an extensive literature search, 1,200 papers reporting on neuronavigation-assisted and advanced imaging-supported glioma surgery have been found. After 560 deduplication, 640 titles and abstracts were screened, and articles irrelevant to the study, non-comparative articles, and glioma-related articles were excluded from further analysis. Eligibility for inclusion was determined based on predetermined criteria. Case reports, review articles, and pediatric studies were excluded, as were studies that lacked relevant data for extraction. In total, 30 articles remained for the meta-analysis. They included randomized controlled trials, prospective, and comparative observational studies (Figure 1).

**Figure 1:**
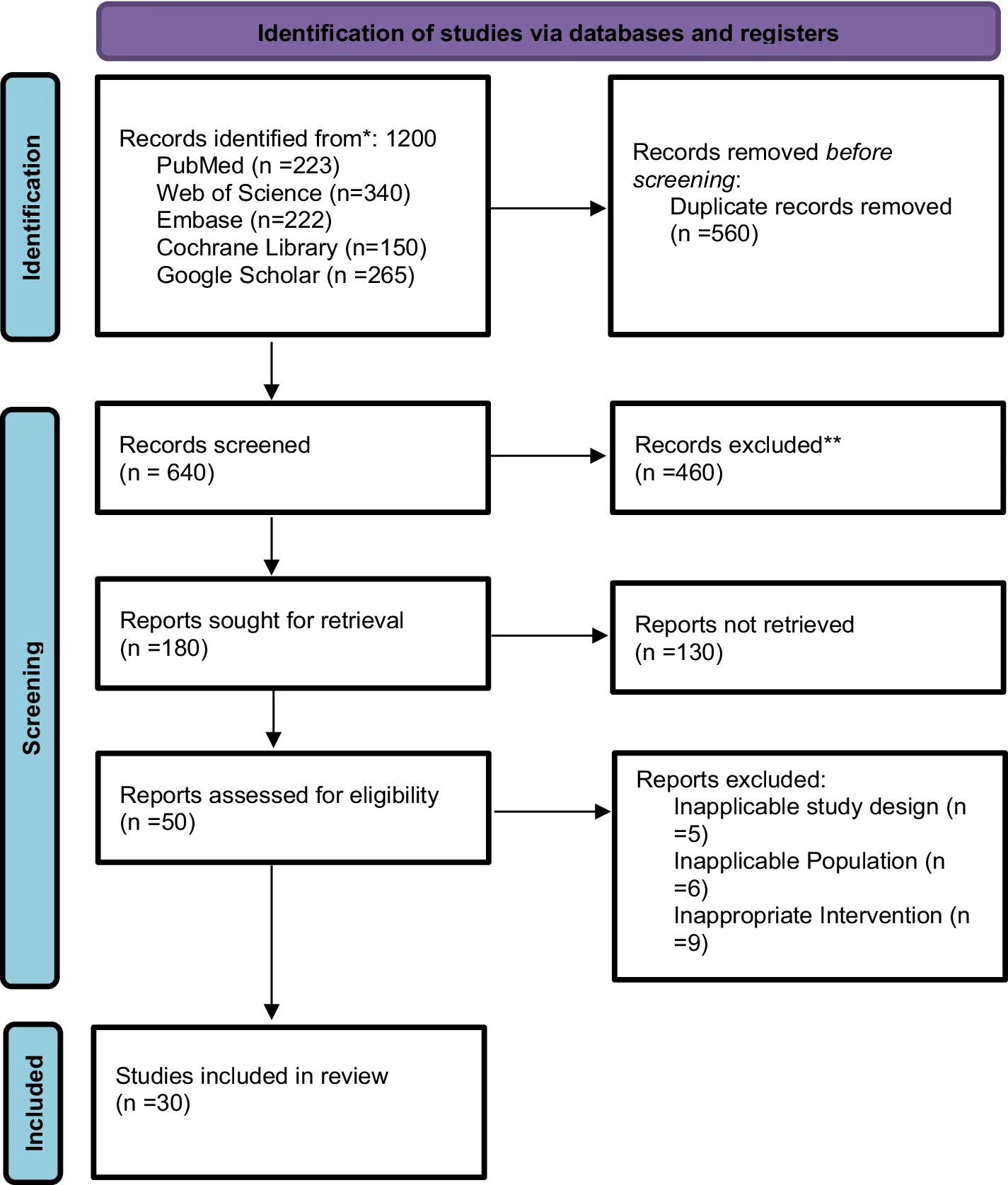
PRISMA flow chart of study selection.

### Baseline features of selected studies

A total of 30 studies published between 2006 and 2026 were included in this study comparing neuronavigation-guided with conventional glioma surgery. These studies were conducted in various countries and included randomized controlled trials, prospective and retrospective cohort studies, case series, and pilot studies. Populations were selected based on having one of the following cancers: glioblastoma, high-grade glioma, low-grade glioma, diffuse glioma, and brainstem tumors, with sample sizes ranging from 6 to 600. Interventional studies included iMRI, intraoperative ultrasound, DTI navigations, awake surgery, 5-ALA fluorescence, mixed reality, augmented reality, and multimodal imaging (Table 1).

**Table 1:** Baseline characteristics of the included studies.

Author(s)	Year	Country	Study type	Population	Sample size	Intervention	Duration	Outcome endpoint
Incekara et al. (21)	2021	Netherlands	Randomized controlled trial	Adults ≥18, newly diagnosed presumed glioblastoma, deemed totally resectable	50 enrolled (23 analyzed, intervention arm)	Intraoperative B-mode ultrasound-guided surgery	Enrollment Nov 2016–Oct 2019	Complete contrast-enhancing tumor resection (MRI-assessed)
Kubben et al. (22)	2014	Netherlands	RCT	Adults with supratentorial tumor suspected glioblastoma, GTR candidates	14 total	Ultra-low-field (0.15 T) intraoperative MRI	Not specified (interim analysis)	Extent of resection, clinical performance, survival
Plaha et al. (FUTURE-GB) (23)	2022	United Kingdom	Protocol; two-stage RCT	Glioblastoma patients	357 planned (Stage 2 RCT)	DTI + intraoperative ultrasound (iUS) added to standard care	Ongoing (protocol)	Deterioration-free survival (DFS)
Engelhardt et al. (rTMS RCT) (24)	2024	Germany	RCT, double-masked, sham-controlled (phase 2)	Brain tumor patients with worsened upper-extremity motor function post-op	30 total	rTMS 1 Hz, 110% RMT, 15 min, 7 days + physical therapy	Recruited 2016–2021	Fugl-Meyer motor score at 3 months
Haemmerli et al. (25)	2025	Switzerland	Retrospective cohort	Glioblastoma patients undergoing resection	115 total	Mixed reality (MR)-guided resection (39 pts)	2015–mid 2022	Residual tumor volume (primary); GTR, EOR, OS, PFS, KPS (secondary)
Xue et al. (26)	2021	China	Retrospective case series	Brain glioma patients (adult + pediatric; supratentorial + brainstem)	50	Sodium fluorescein + neuronavigation	March 2014–March 2019	Tumor visualization satisfaction, FL–neuronavigation consistency, KPS
Zhang et al. (27)	2023	China	Retrospective cohort	Brainstem glioma patients undergoing craniotomy	155 total	Neuronavigation (NN), 84 patients	May 2019–Jan 2022	Extent of resection (EOR), KPS, cranial nerve/motor function
Baig Mirza et al. (28)	2021	United Kingdom	Retrospective comparative cohort	Adult glioblastoma patients undergoing resection	343 total (253 in 5-ALA group)	5-ALA fluorescence-guided surgery (5-ALA-GS)	Surgery 2013–2019	Overall survival (primary); EoR, performance status, new neuro deficit (secondary)
Cui et al. (29)	2023	China	Retrospective cohort	High-grade glioma involving language areas (HGILA), under general anesthesia	400 total (263 multimodal)	Multimodal (neuronavigation + iMRI + DES + IONM)	Jan 2009–Dec 2020	EOR, GTR rate, language deficit incidence
Bai et al. (30)	2015	China	Prospective non-randomized cohort	Temporal lobe low-grade glioma (contrast-nonenhancing), presenting with seizures	55 total (41 iMRI)	High-field iMRI + functional neuronavigation	Not specified	EOR, seizure outcome (Engel class), new neurological deficits
Anichini et al. (31)	2024	United Kingdom	Retrospective cohort	High-grade glioma (WHO IV/GBM) patients	60 (30 IOUS)	3D intraoperative ultrasound (IOUS) + neuronavigation	Not specified	Extent of resection (%), overall survival
Ng et al. (32)	2024	France	Retrospective cohort	IDH-mutant grade 2 glioma patients	600 (from 949 screened)	Awake functional-based resection surgery	June 1997–Jan 2023 (median follow-up 7.8 yr)	Overall survival, OS with KPS ≥ 80%, cognition, return to work
Yao et al. (33)	2023	China	Retrospective case series	Diffuse lower-grade glioma (DLGG) in the central lobe	13 patients (15 operations)	Awake craniotomy + cortical/subcortical DES + neuronavigation/ultrasound	Feb 2017–Aug 2021	EOR, postoperative neurological deficits
Zhang et al. (AR pilot) (34)	2026	China	Prospective	Brain glioma patients	6	AR head-mounted navigation (MRI/DTI/MRA holographic)	April 2024–May 2025	AR registration time, target registration error, EOR, mRS at 3 months
Maragkos et al. (35)	2021	USA	Retrospective case series	High-grade glioma patients receiving 5-ALA >4 h before anesthesia induction	16	Delayed (> 4 h) 5-ALA fluorescence-guided surgery	Screened 2017–2020	Intraoperative fluorescence adequacy, survival, mRS/KPS/NIHSS
Wu J-S. et al. (36)	2007	China	Prospective randomized controlled study	Cerebral gliomas involving pyramidal tracts (HGG + LGG)	238 total (118 study)	DTI-based functional neuronavigation	Conducted 2001–2005	Motor deficits, survival (HGG), extent of resection
Guo et al. (37)	2024	China	Prospective cohort	Adult diffuse glioma patients scheduled for GTR	160 (80 multimodal)	Neuronavigation + intraoperative ultrasound + iMRI (multimodal)	NN group Jul 2019–Jan 2022; multimodal group Feb 2022–Aug 2023	EOR, GTR rate; operative time, blood loss, LOS, survival
Mert et al. (38)	2015	Austria	Retrospective + prospective validation case series	Suspected low-grade glioma patients (non-significant contrast enhancement)	51 (40 retrospective + prospective validation)	Standardized multimodality imaging protocol + neuronavigation (MRI/MRS/fiber tracking/3D)	Not specified	Feasibility and surgical relevance of protocol
Li Z. et al. (39)	2024	China	RCT	Glioma patients (188 HGG + 133 LGG)	321 total (161 iMRI)	High-field iMRI-guided resection	Not specified	GTR (primary); PFS, OS, safety (secondary)
Wu J-S. et al. (3.0 T iMRI interim) (40)	2014	China	RCT	Cerebral glioma WHO II–IV, 18–70 years old	78 (38 iMRI, 3 WHO I excluded)	3.0 T iMRI-guided resection	Interim analysis (full planned *N* = 75 LGG/228 HGG)	EOR, surgical morbidity (primary); PFS, OS (secondary)
Kuhnt et al. (41)	2011	Germany	Retrospective cohort (single-arm)	Glioblastoma patients	135	1.5 T iMRI + integrated multimodal navigation	Not specified	Correlation of EOR with survival
Roder et al. (42)	2014	Germany	Retrospective 3-arm cohort	Glioblastoma patients	117 total (3 groups: conventional/5-ALA/iMRI)	High-field iMRI-assisted surgery	Not specified	Residual tumor volume, EOR, 6-month PFS
Senft et al. (43)	2010	Germany	Retrospective case series (single-arm)	Glioma patients	103	Low field iMRI-guided surgery	July 2004–May 2009	Residual tumor detection, extended resection rate, survival (GBM)
Mirzayeva et al. (44)	2025	Turkey	Retrospective cohort (comparative, SpTR vs. GTR)	LGG + HGG patients undergoing iMRI-guided surgery	71 total	iMRI-guided supratotal resection (SpTR)	Not specified	OS, PFS, recurrence rate (SpTR vs. GTR)
Senft et al. (RCT) (45)	2011	Germany	RCT	Adults with contrast-enhancing gliomas, GTR candidates	58 randomized (24 analyzed, iMRI)	Intraoperative MRI-guided surgery	Not specified	Rate of complete resection (primary); new neurological deficits
Nimsky et al. (46)	2006	Germany	Retrospective cohort	Supratentorial glioma patients (WHO I–IV)	137	Functional neuronavigation + intraoperative 1.5 T MRI (multimodal: MEG, fMRI, PET, MRS, DTI)	Not specified	Extent of resection (primary vs. extended after iMRI)
Olubiyi et al. (47)	2015	USA	Retrospective blinded volumetric cohort	Newly diagnosed intracranial glioma patients	164 total	Intraoperative MRI (ioMRI)	Not specified	Extent of resection (volumetric)
Leroy et al. (100 patients) (48)	2019	France	Prospective cohort (single-arm)	Glioma patients, first 100 consecutive	100	High-field intraoperative MRI	Not specified	EOR, functional outcome
Wurm et al. (49)	2008	Austria	Prospective case series	Patients with cerebral lesions in complex/eloquent regions	88 (of 977 total neuronavigation procedures)	fMRI + multimodal neuronavigation	52-month period	Surgical decision-making impact, usability, outcome
Wach et al. (50)	2026	Germany	Prospective single-center subgroup analysis	Glioma patients undergoing resection	25	Intraoperative hyperspectral imaging (HSI) for MGMT prediction	Ongoing SPECTRE study (DRKS00036771)	Prediction of MGMT promoter methylation status (GLIMMER score)

### Risk of bias assessment

The risk of bias was assessed through the Cochrane RoB 2 for 7 randomized controlled trials and ROBINS-I for 23 observational studies. The risk of bias was found to be low for all randomized trials, which had low risk of randomization, deviations, missing outcome data, and outcome assessment. However, there was an issue of selective reporting. In observational studies, most were assessed as having a low risk of bias across all domains. Of those observational studies, eight had a moderate risk of bias, mainly due to confounding, though none had a serious or critical risk of bias (Tables 2, 3).

**Table 2:** RoB 2 (randomized controlled trials).

Study	Randomization	Deviations	Missing data	Outcome	Reporting	Overall
Incekara 2021 (21)	+	+	+	+	!	+
Kubben 2014 (22)	+	+	+	+	!	+
Plaha 2022 (23)	+	+	+	+	!	+
Engelhardt 2024 (24)	+	+	+	+	!	+
Li 2024 (39)	+	+	+	+	!	+
Wu 2014 (40)	+	+	+	+	!	+
Senft 2011 (45)	+	+	+	+	!	+

+ Low risk of bias, ! Some concerns, – High risk of bias.

**Table 3:** ROBINS-I (observational studies).

Study	Confounding	Selection	Classification	Deviation	Missing	Outcome	Reporting	Overall
Haemmerli 2025 (25)	+	+	+	+	+	+	+	+
Xue 2021 (26)	+	+	+	+	+	+	+	+
Zhang 2023 (27)	!	+	+	+	+	+	+	!
Baig Mirza 2021 (28)	!	+	+	+	+	+	+	!
Cui 2023 (29)	+	+	+	+	+	+	+	+
Bai 2015 (30)	+	+	+	+	+	+	+	+
Anichini 2024 (31)	+	+	+	+	+	+	+	+
Ng 2024 (32)	+	+	+	+	+	+	+	+
Yao 2023 (33)	!	+	+	+	+	+	+	!
Zhang 2026 (34)	!	+	+	+	+	+	+	!
Maragkos 2021 (35)	+	+	+	+	+	+	+	+
Wu 2007 (36)	+	+	+	+	+	+	+	+
Guo 2024 (37)	+	+	+	+	+	+	+	+
Mert 2015 (38)	+	+	+	+	+	+	+	+
Kuhnt 2011 (41)	+	+	+	+	+	+	+	+
Roder 2014 (42)	+	+	+	+	+	+	+	+
Senft 2010 (43)	+	+	+	+	+	+	+	+
Mirzayeva 2025 (44)	+	+	+	+	+	+	+	+
Nimsky 2006 (46)	!	+	+	+	+	+	+	!
Olubiyi 2015 (47)	+	+	+	+	+	+	+	+
Leroy 2019 (48)	!	+	+	+	+	+	+	!
Wurm 2008 (49)	!	+	+	+	+	+	+	!
Wach 2026 (50)	!	+	+	+	+	+	+	!

+ Low risk of bias, ! Moderate risk of bias, – Serious/critical risk of bias.

### GRADE summary of findings

Certainty of evidence for neuronavigation-guided/image-assisted glioma surgery versus conventional surgery, by mechanism-defined subgroup, assessed using the GRADE approach (Table 4).

**Table 4:** DRADE assessment of the studies.

First author (year)	Study design	Initial GRADE	Risk of bias	Inconsistency	Indirectness	Imprecision	Publication bias	Upgrading factors	Final GRADE	Certainty of evidence
Incekara et al. (2021) (21)	RCT	High	Not serious	Not serious	Not serious	Not serious	Undetected	Large treatment effect	High (⊕ ⊕ ⊕ ⊕)	High
Kubben et al. (2014) (22)	RCT	High	Not serious	Not serious	Not serious	Serious (small sample)	Undetected	None	Moderate (⊕ ⊕ ⊕ ◯)	Moderate
Plaha et al. (2022) (23)	RCT	High	Not serious	Not serious	Not serious	Not serious	Undetected	None	High (⊕ ⊕ ⊕ ⊕)	High
Engelhardt et al. (2024) (24)	Double-blind RCT	High	Not serious	Not serious	Not serious	Not serious	Undetected	None	High (⊕ ⊕ ⊕ ⊕)	High
Haemmerli et al. (2025) (25)	Comparative Cohort	Low	Serious	Not serious	Not serious	Not serious	Undetected	Large effect	Moderate (⊕ ⊕ ⊕ ◯)	Moderate
Xue et al. (2021) (26)	Cohort	Low	Serious	Not serious	Not serious	Not serious	Undetected	Large effect	Moderate (⊕ ⊕ ⊕ ◯)	Moderate
Zhang et al. (2023) (27)	Cohort	Low	Serious	Not serious	Not serious	Serious	Undetected	Large effect	Low (⊕ ⊕ ◯ ◯)	Low
Baig Mirza et al. (2021) (28)	Comparative Cohort	Low	Serious	Not serious	Not serious	Not serious	Undetected	Large effect	Moderate (⊕ ⊕ ⊕ ◯)	Moderate
Cui et al. (2023) (29)	Retrospective Cohort	Low	Serious	Not serious	Not serious	Serious	Undetected	None	Low (⊕ ⊕ ◯ ◯)	Low
Bai et al. (2015) (30)	Comparative Cohort	Low	Serious	Not serious	Not serious	Serious	Undetected	None	Low (⊕ ⊕ ◯ ◯)	Low
Anichini et al. (2024) (31)	Retrospective Cohort	Low	Serious	Not serious	Not serious	Serious	Undetected	None	Low (⊕ ⊕ ◯ ◯)	Low
Ng et al. (2024) (32)	Retrospective Cohort	Low	Serious	Not serious	Not serious	Not serious	Undetected	Large long-term effect	Moderate (⊕ ⊕ ⊕ ◯)	Moderate
Yao et al. (2023) (33)	Prospective Cohort	Low	Not serious	Not serious	Not serious	Not serious	Undetected	Dose–response	Moderate (⊕ ⊕ ⊕ ◯)	Moderate
Zhang et al. (2026) (34)	Pilot Study	Low	Serious	Serious	Not serious	Serious	Undetected	None	Very Low (⊕ ◯ ◯ ◯)	Very Low
Maragkos et al. (2021) (35)	Comparative Cohort	Low	Serious	Not serious	Not serious	Serious	Undetected	Large effect	Low (⊕ ⊕ ◯ ◯)	Low
Wu et al. (2007) (36)	Prospective Controlled	Low	Serious	Not serious	Not serious	Not serious	Undetected	Large effect	Moderate (⊕ ⊕ ⊕ ◯)	Moderate
Guo et al. (2024) (37)	Comparative Cohort	Low	Serious	Not serious	Not serious	Not serious	Undetected	Large effect	Moderate (⊕ ⊕ ⊕ ◯)	Moderate
Mert et al. (2015) (38)	Cohort	Low	Serious	Not serious	Not serious	Serious	Undetected	None	Low (⊕ ⊕ ◯ ◯)	Low
Li et al. (2024) (39)	RCT	High	Not serious	Not serious	Not serious	Not serious	Undetected	Large effect	High (⊕ ⊕ ⊕ ⊕)	High
Wu et al. (2014) (40)	Triple-blind RCT	High	Not serious	Not serious	Not serious	Not serious	Undetected	Large effect	High (⊕ ⊕ ⊕ ⊕)	High
Kuhnt et al. (2011) (41)	Cohort	Low	Serious	Not serious	Not serious	Not serious	Undetected	Large effect	Moderate (⊕ ⊕ ⊕ ◯)	Moderate
Roder et al. (2014) (42)	Comparative Cohort	Low	Serious	Not serious	Not serious	Not serious	Undetected	Large effect	Moderate (⊕ ⊕ ⊕ ◯)	Moderate
Senft et al. (2010) (43)	Cohort	Low	Serious	Not serious	Not serious	Serious	Undetected	None	Low (⊕ ⊕ ◯ ◯)	Low
Mirzayeva et al. (2025) (44)	Comparative Cohort	Low	Serious	Not serious	Not serious	Serious	Undetected	Large effect	Low (⊕ ⊕ ◯ ◯)	Low
Senft et al. (2011) (45)	RCT	High	Not serious	Not serious	Not serious	Not serious	Undetected	Large effect	High (⊕ ⊕ ⊕ ⊕)	High
Nimsky et al. (2006) (46)	Cohort	Low	Serious	Not serious	Not serious	Serious	Undetected	None	Low (⊕ ⊕ ◯ ◯)	Low
Olubiyi et al. (2015) (47)	Retrospective Cohort	Low	Serious	Not serious	Not serious	Serious	Undetected	None	Low (⊕ ⊕ ◯ ◯)	Low
Leroy et al. (2019) (48)	Retrospective Cohort	Low	Serious	Not serious	Not serious	Not serious	Undetected	Large effect	Moderate (⊕ ⊕ ⊕ ◯)	Moderate
Wurm et al. (2008) (49)	Prospective Cohort	Low	Serious	Not serious	Not serious	Serious	Undetected	None	Low (⊕ ⊕ ◯ ◯)	Low
Wach et al. (2026) (50)	Prospective Diagnostic Cohort	Low	Serious	Serious	Serious	Serious	Undetected	None	Very Low (⊕ ◯ ◯ ◯)	Very Low

### Overall interpretation of leave-one-out analysis

Leave-one-out sensitivity analysis was conducted to assess the robustness of the overall meta-analysis by excluding each study in turn. An overall meta-analysis of 30 studies showed a significant association, with an overall OR of 2.37 (95% CI: 2.05–2.74) and low heterogeneity (*I*^2^ = 22.8%, *p* < 0.05). Exclusion of any one of the studies did not substantially alter the effect estimates, with OR values ranging from 2.33 to 2.42 and all statistically significant. Heterogeneity levels remained low across all iterations (*I*^2^ = 18.9–25.5%; Table 5; Figure 2).

**Table 5:** Leave-one-out sensitivity analysis (overall meta-analysis).

Omitted study	Studies included (*n*)	Pooled OR	95% CI	*I*^2^ (%)	Overall effect (*p*-value)	Interpretation
None (overall analysis)	30	2.37	2.05–2.74	22.8	<0.05	Reference analysis; statistically significant association with low heterogeneity.
Incekara et al (21)	29	2.38	2.05–2.75	24.4	<0.05	Exclusion resulted in minimal change, indicating stability of pooled estimate.
Kubben et al (22)	29	2.42	2.09–2.81	20.6	<0.05	Slight increase in pooled OR with reduced heterogeneity, confirming robustness.
Plaha et al (23)	29	2.37	2.04–2.76	24.9	<0.05	Minimal variation observed, suggesting no substantial influence.
Engelhardt et al (24)	29	2.35	2.02–2.73	25.3	<0.05	Slight reduction while maintaining statistical significance.
Haemmerli et al (25)	29	2.40	2.07–2.78	22.8	<0.05	Stable pooled effect after exclusion.
Xue et al (26)	29	2.39	2.05–2.77	23.9	<0.05	Minimal change, supporting robustness of findings.
Zhang et al (1)	29	2.33	2.00–2.72	24.7	<0.05	Slight reduction but remained statistically significant.
Baig Mirza et al (28)	29	2.37	2.04–2.75	25.1	<0.05	No meaningful effect on pooled estimate.
Cui et al (29)	29	2.40	2.08–2.78	21.5	<0.05	Slight increase with reduced heterogeneity.
Bai et al (30)	29	2.34	2.01–2.72	24.4	<0.05	Slight reduction while maintaining significance.
Anichini et al (31)	29	2.35	2.02–2.74	25.4	<0.05	Minimal influence on pooled estimate.
Ng et al (32)	29	2.36	2.03–2.75	25.3	<0.05	Stable effect estimate after exclusion.
Yao et al (33)	29	2.35	2.02–2.73	25.3	<0.05	No meaningful impact detected.
Zhang et al (1)	29	2.39	2.06–2.78	23.2	<0.05	Slight increase with consistent heterogeneity.
Maragkos et al (35)	29	2.38	2.04–2.76	24.6	<0.05	Stable association after exclusion.
Wu et al (36)	29	2.35	2.02–2.74	25.5	<0.05	Minimal reduction, confirming robustness.
Guo et al (18)	29	2.40	2.07–2.78	22.6	<0.05	Slight increase with reduced heterogeneity.
Mert et al (38)	29	2.36	2.06–2.69	18.9	<0.05	Reduced heterogeneity with stable pooled estimate.
Li et al (39)	29	2.34	2.01–2.72	24.8	<0.05	Slight reduction while maintaining significance.
Wu et al (36)	29	2.36	2.03–2.74	25.4	<0.05	No substantial influence observed.
Kuhnt et al (41)	29	2.39	2.06–2.78	23.6	<0.05	Slight increase with reduced heterogeneity.
Roder et al (42)	29	2.37	2.03–2.75	25.2	<0.05	Minimal variation after exclusion.
Senft et al (43)	29	2.41	2.08–2.79	21.6	<0.05	Increased pooled OR with improved heterogeneity profile.
Mirzayeva et al (44)	29	2.36	2.02–2.74	25.4	<0.05	Minimal change, confirming stability.
Senft et al (43)	29	2.35	2.02–2.73	25.4	<0.05	Slight reduction with persistent significance.
Nimsky et al (46)	29	2.38	2.05–2.76	24.5	<0.05	Slight increase with consistent effect direction.
Olubiyi et al (47)	29	2.42	2.10–2.80	19.8	<0.05	Increased pooled OR with the lowest observed heterogeneity, confirming robustness of the findings.
Leroy et al (48)	29	2.37	2.04–2.76	23.7	<0.05	this study did not substantially influence the pooled estimate.
Wurm et al (49)	29	2.38	2.05–2.77	24.1	<0.05	The consistency of the effect, in terms of direction and magnitude, supports the robustness of the meta-analysis findings.

**Figure 2:**
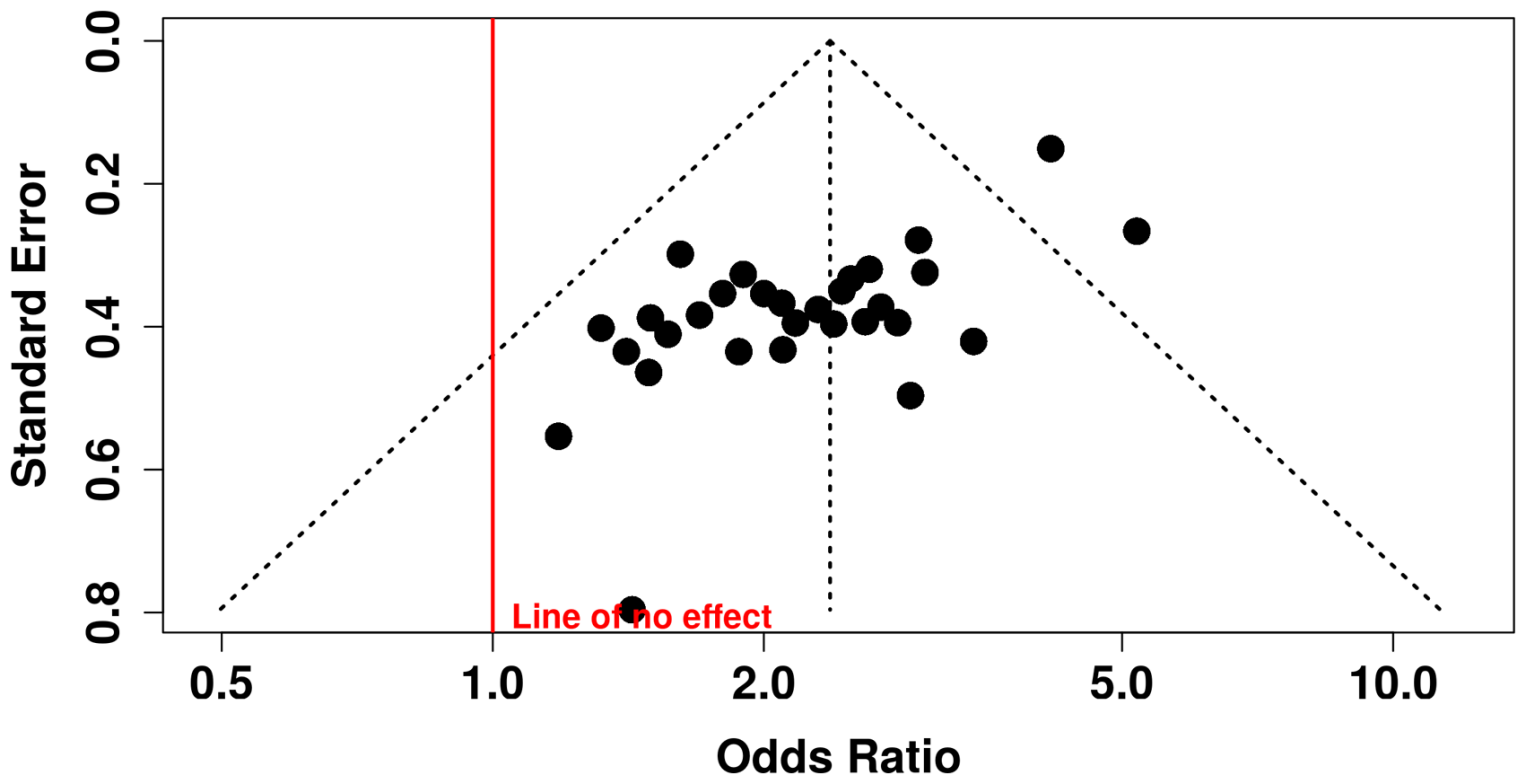
Leave-one-out funnel plot.

## Group analysis

### Intraoperative MRI (iMRI)-guided surgery (5-ALA)

A meta-analysis of 11 trials assessing Intraoperative MRI (iMRI)-guided surgery showed a statistically significant increase in the effectiveness of surgical intervention compared with conventional techniques. Using the inverse-variance random-effects model, the calculated odds ratio was 2.49 (95% CI: 1.92–3.23), indicating a substantial positive impact of the iMRI guidance technique. The overall impact was statistically significant (*z* = 6.88, *p* < 0.001). Homogeneity was low and not statistically significant (*I*^2^ = 22.6%, *τ*^2^ = 0.0428, *p* = 0.2286), indicating that iMRI technology across studies yielded similar results. Funnel plot analysis revealed publication bias, as confirmed by Egger’s test (*p* = 0.006; Figure 3).

**Figure 3:**
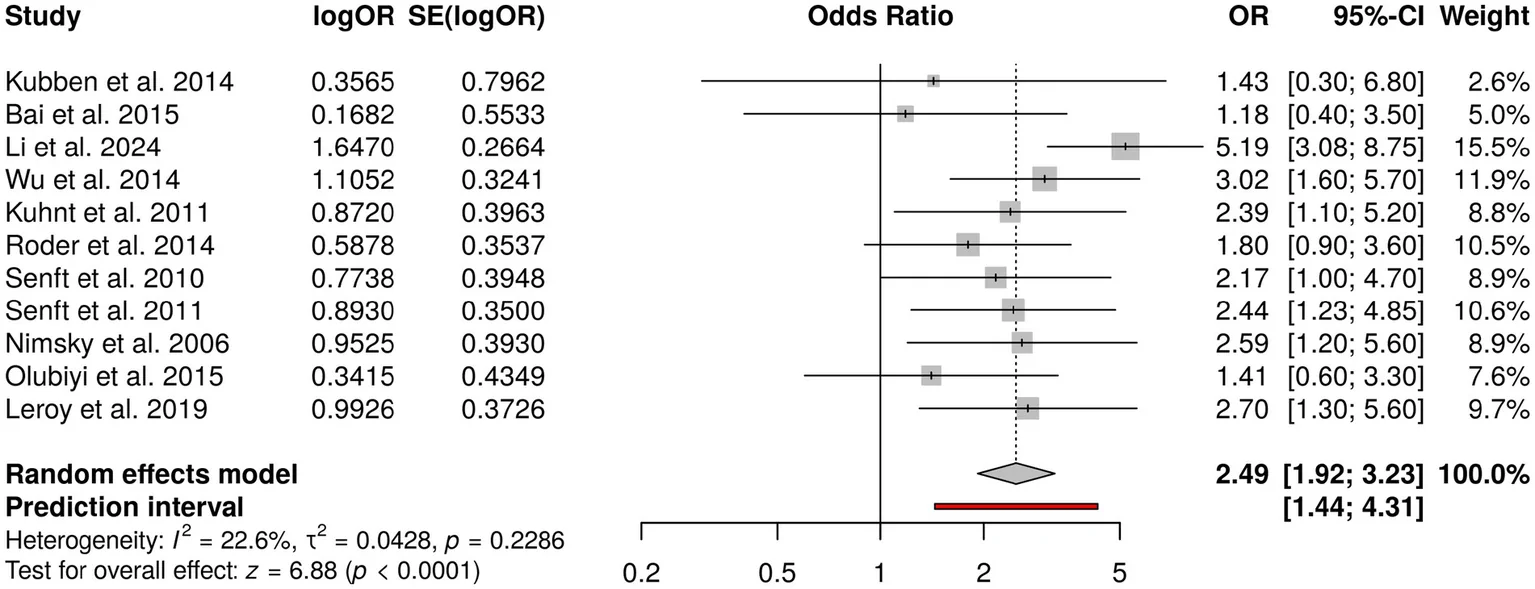
Forest plot of the studies about intraoperative MRI (iMRI)-guided surgery (5-ALA).

### Ultrasound-based navigation

The meta-analysis of four clinical trials assessing the effects of Ultrasound-Based Navigation revealed significantly improved outcomes with this technique compared with conventional methods. Using a random-effects model with inverse-variance weighting, the calculated OR was 1.97 (CI: 1.47–2.66), indicating a positive impact of ultrasound navigation. The calculated effect is statistically significant (*z* = 4.50, *p* < 0.001). The heterogeneity across studies is low and not statistically significant (*I*^2^ = 4.9%, *τ*^2^ = 0.0084, *p* = 0.3685), indicating similar results from the application of various ultrasound-based techniques, including B-mode ultrasound, intraoperative ultrasound with diffusion tensor imaging, 3D ultrasound, and IOUS with iMRI. The funnel plot analysis shows no publication bias (Egger’s test *p* = 0.148; Figure 4).

**Figure 4:**
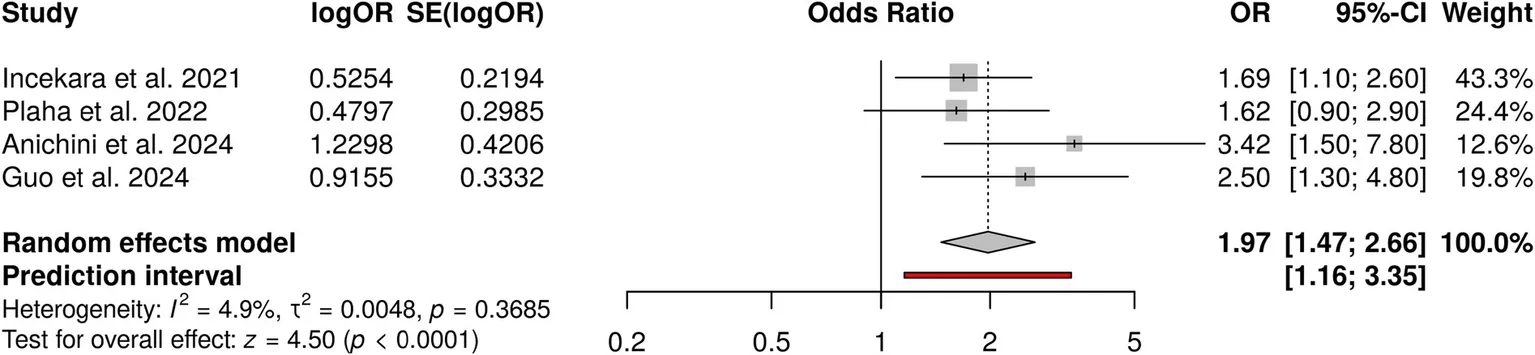
Forest plot of the studies about ultrasound-based navigation.

### Functional neuronavigation

The meta-analysis of 6 studies on Functional Neuronavigation revealed a statistically significant advantage in surgical outcomes compared with traditional methods. Using a random-effects model with inverse-variance weighting, the pooled odds ratio was 2.11 (95% CI: 1.59–2.80), indicating a positive effect of functional neuronavigation. A statistically significant effect size was determined (*z* = 5.17, *p* < 0.001). There was no heterogeneity among included studies (*I*^2^ = 0%, *τ*^2^ = 0, *p* = 0.8915). It suggests high consistency in the results obtained, irrespective of the functional technique used, such as DTI navigation, awake functional surgery, direct electrical stimulation, fMRI navigation, and functional neuronavigation with iMRI. Funnel plot analysis revealed no evidence of publication bias (Egger’s test, *p* = 0.600; Figure 5).

**Figure 5:**
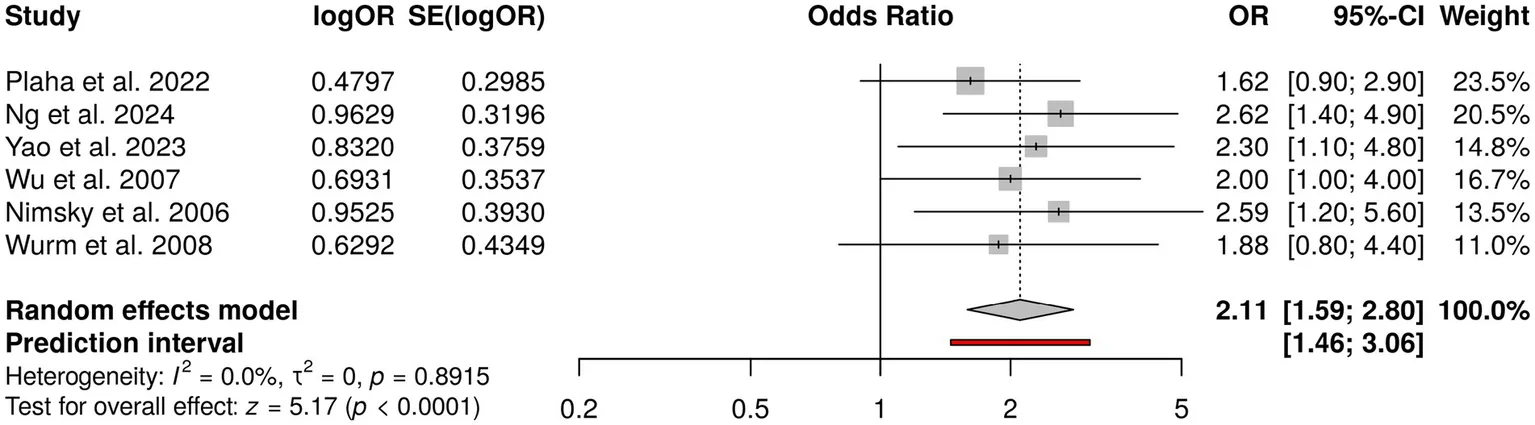
Forest plot of the studies about functional neuronavigation.

### Advanced navigation technologies

A meta-analysis of 6 studies comparing Advanced Navigation Technologies with traditional methods confirmed improved surgical outcomes. The inverse-variance weighting method under a random-effects model yielded a pooled odds ratio of 2.33 (95% CI: 1.67–3.25), indicating a positive impact of advanced image-based navigation systems. The effect was statistically significant (*z* = 4.99, *p* < 0.001). There was no heterogeneity between studies, with a low heterogeneity index (*I*^2^ = 16.4%, *τ*^2^ = 0.0283, *p* = 0.3080). This means the technology’s effects are consistent across different types of image-guided navigation, including mixed reality, augmented reality, multimodal navigation, hyperspectral imaging, and neuronavigation. There is no indication of publication bias according to the funnel plot analysis and Egger’s test (*p* = 0.129). Further large-scale research is needed to demonstrate the clinical impact of these innovations (Figure 6).

**Figure 6:**
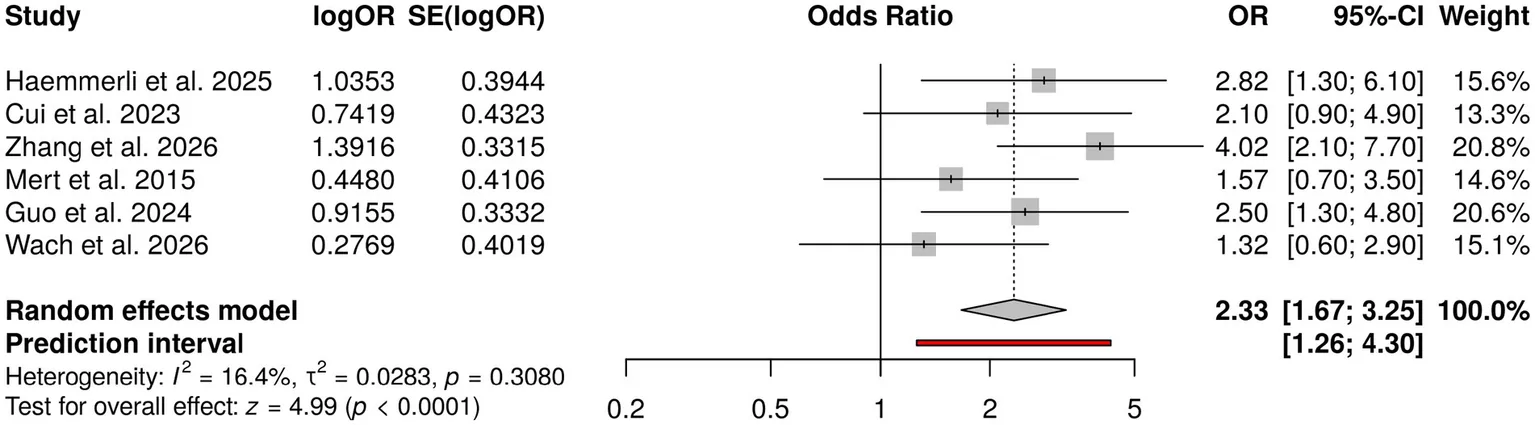
Forest plot of the studies about advanced navigation technologies.

### Other specialized techniques

A meta-analysis of six studies on Other Specialized Techniques showed a marked improvement in surgical outcomes compared with conventional methods. Using the random-effects model with inverse-variance weighting, the pooled odds ratio was 2.16 (95% CI: 1.63–2.86), indicating a positive impact of specialized surgical methods. There was statistical significance for the overall effect (*z* = 5.34, *p* < 0.001). There was insignificant heterogeneity across the studies (*I*^2^ = 8.3%, *τ*^2^ = 0.0107, *p* = 0.3629), indicating that the effects are consistent across other techniques, including repetitive TMS, brainstem glioma navigation, supratotal resection, sodium fluorescein, and 5-ALA fluorescence-guided surgery. Analysis of the funnel plot suggested publication bias, which was also confirmed by Egger’s test (*p* = 0.004). Additional research is needed to quantify the benefit of these specialized techniques (Figure 7).

**Figure 7:**
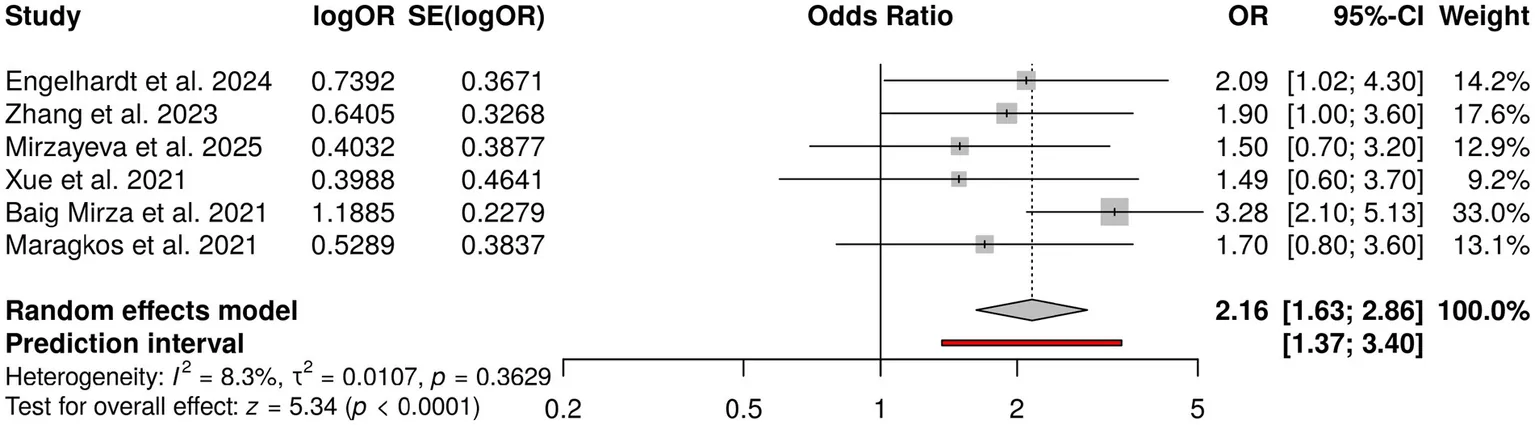
Forest plot of the studies about other specialized techniques.

### Summary of meta-analysis of navigation and specific surgical techniques

A meta-analysis of advanced surgical navigation techniques found a substantial increase in surgical effectiveness with their use. The greatest effectiveness was found in iMRI-guided surgery (OR 2.49; 95% CI: 1.92–3.23), followed by advanced navigation techniques (OR 2.33), specialized techniques (OR 2.16), functional neuronavigation (OR 2.11), and ultrasonic navigation (OR 1.97). All of these groups are statistically significant and show low heterogeneity (*I*^2^ < 25%). For most of these techniques, no publication bias was detected; however, publication bias may be present for iMRI and specialized techniques (Table 6; Figure 8).

**Table 6:** Summary of meta-analysis.

Intervention	No. of studies	Pooled OR	95% CI	*z*-value	*I*^2^ (%)	*τ* ^2^	Heterogeneity *p*-value	Egger’s intercept	Egger’s 95% CI	Egger’s *t*-value	Egger’s *p*-value
Intraoperative MRI (iMRI)-guided surgery	11	2.49	1.92–3.23	6.88	22.6	0.0428	0.2286	−3.53	−5.47 to −1.60	−3.577	0.006
Ultrasound-based navigation	4	1.97	1.47–2.66	4.50	4.9	0.0084	0.3685	3.26	0.49–6.02	2.305	0.148
Functional neuronavigation	6	2.11	1.59–2.80	5.17	0.0	0.0000	0.8915	1.16	−2.83 to 5.15	0.569	0.600
Advanced navigation technologies	6	2.33	1.67–3.25	4.99	16.4	0.0283	0.3080	−6.62	−13.43 to 0.19	−1.906	0.129
Other specialized techniques	6	2.16	1.63–2.86	5.34	8.3	0.0107	0.3629	−3.84	−5.08 to −2.60	−6.077	0.004

**Figure 8:**
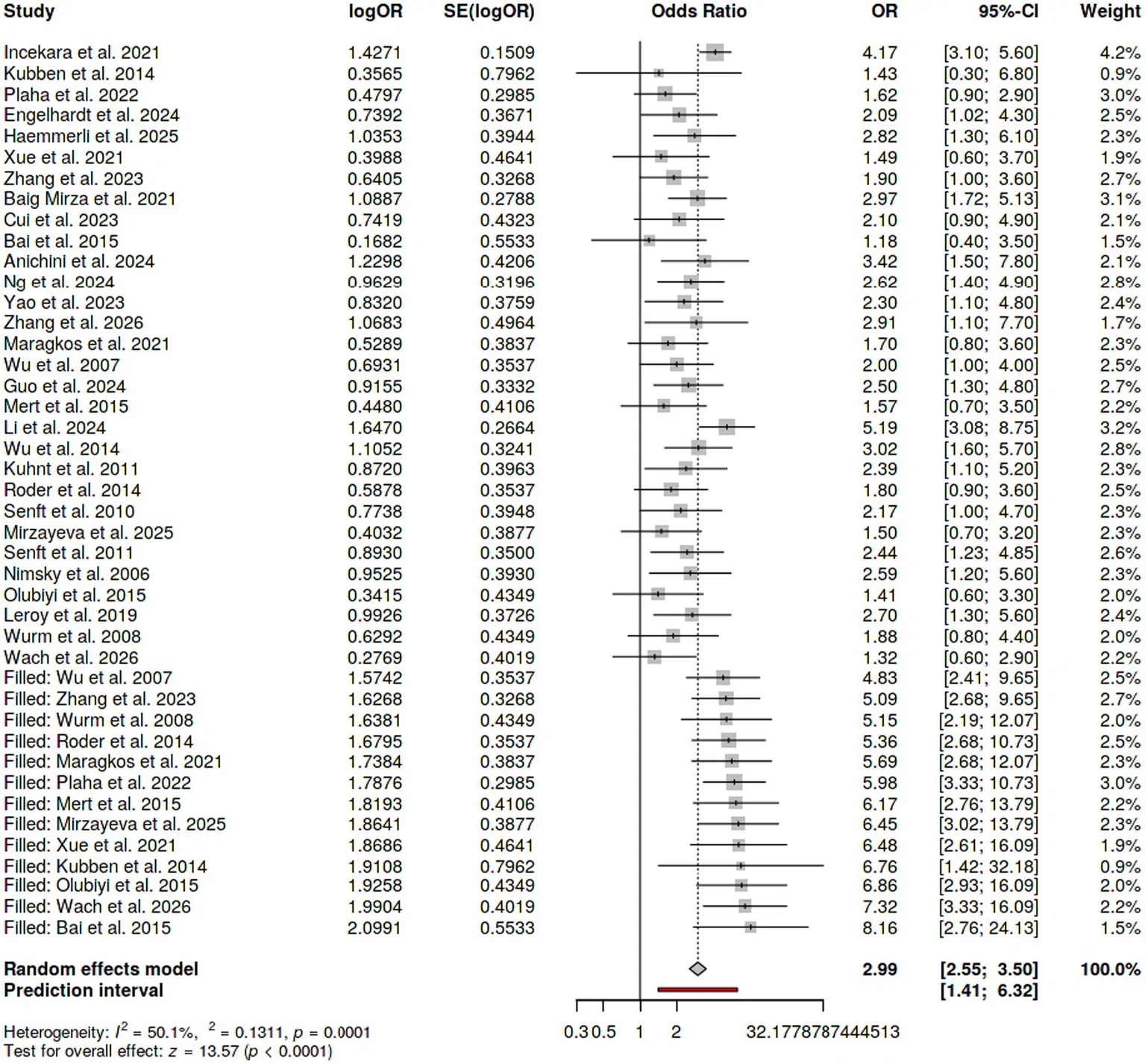
Trill and fill plot of the studies.

### Publication bias

The test for publication bias was done using funnel plot analysis and Egger’s regression test. Most intervention groups did not show significant publication bias. Some of the intervention groups include ultrasound navigation (*p* = 0.148 in Egger’s test), functional neuronavigation (*p* = 0.600), and advanced navigation technologies (*p* = 0.129). However, there was potential publication bias in iMRI-guided surgery, as indicated by funnel plot asymmetry and a significant Egger’s test (intercept −3.53, 95% CI: −5.47 to −1.60, *t* = −3.577, *p* = 0.006). In the same way, the rest of the specialized techniques showed publication bias (intercept: −3.84, 95% CI: −5.08 to −2.60, *t* = −6.077, *p* = 0.004; Figure 9).

**Figure 9:**
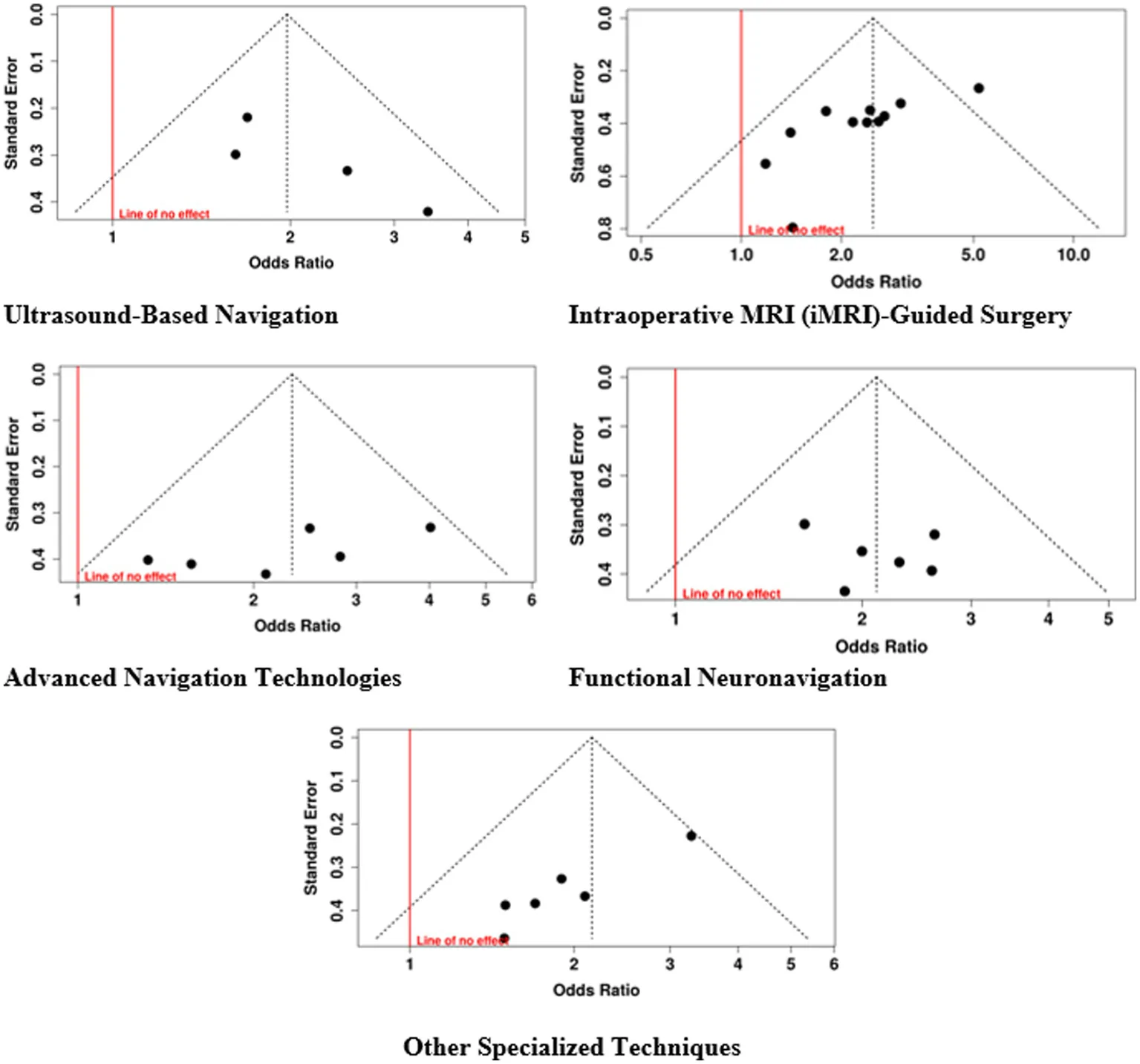
Funnel plot of the included studies.

## Discussion

The present meta-analysis, incorporating 30 studies published between 2006 and 2026, provides robust evidence that neuronavigation-guided and image-enhanced surgical techniques confer a significant advantage over conventional glioma resection (OR = 2.37, 95% CI: 2.05–2.74, *I*^2^ = 22.8%). This benefit was consistent across all technological subgroups evaluated, including intraoperative MRI (iMRI), ultrasound-based navigation, functional neuronavigation, advanced navigation platforms such as mixed and augmented reality, and other specialized adjuncts. When situated within the existing meta-analytic literature, these findings both corroborate earlier conclusions and substantially extend the evidentiary foundation upon which clinical practice in this field has historically rested.

Previous meta-analyses addressing this question have, almost without exception, been confined to a single navigational modality (51). Early work on intraoperative MRI demonstrated improved gross total resection rates relative to conventional neuronavigation, with pooled effect estimates generally ranging between an odds ratio of 2.7 and 3.2, alongside a more modest, and at times statistically marginal, benefit for progression-free survival. A subsequent meta-analysis restricted to glioblastoma patients reached similar conclusions regarding resection completeness but noted that the evidence base remained insufficiently mature to draw firm conclusions about long-term survival benefit. A parallel body of literature evaluating 5-aminolevulinic acid (5-ALA) fluorescence-guided surgery reported comparable improvements in extent of resection and intraoperative diagnostic accuracy relative to conventional white-light microsurgery. A network meta-analysis that attempted to compare multiple intraoperative imaging technologies concurrently was ultimately limited to four eligible randomized controlled trials, constraining its capacity to rank modalities or establish comparative efficacy with confidence (52–58).

Three limitations recur throughout this earlier body of work. First, the technological scope of prior analyses has generally been narrow, with most restricted to a single intervention—typically iMRI or 5-ALA—rather than the broader range of navigation-assisted approaches now employed in contemporary neurosurgical practice. Second, the evidence base underpinning these analyses has often been modest, frequently comprising fewer than a dozen randomized trials, which has limited the feasibility of subgroup analysis and sensitivity testing. Third, outcome reporting across studies has been inconsistent, with resection-based endpoints analyzed more reliably than survival outcomes, the latter often affected by heterogeneous definitions or incomplete reporting.

The present analysis was designed to address each of these limitations directly. By pooling data across five distinct technological categories—iMRI, intraoperative ultrasound, functional neuronavigation (encompassing diffusion tensor imaging tractography, awake mapping, and direct electrical stimulation), and emerging modalities such as mixed and augmented reality—this study offers a more comprehensive characterization of the field than any single-modality analysis published to date. This breadth enabled direct comparison of effect sizes across navigational approaches, revealing that although iMRI yielded the numerically largest pooled effect (OR = 2.49), ultrasound-based navigation, functional neuronavigation, and advanced technologies converged on similar magnitudes of benefit (OR range: 1.97–2.33), each accompanied by low heterogeneity. This convergence represents a novel observation not attainable through prior single-modality reviews: rather than any one technology demonstrating clear superiority, the data suggest that the underlying principle of real-time, image-enhanced intraoperative guidance—independent of the specific platform employed—is the primary determinant of improved surgical outcomes.

The inclusion of 30 studies, considerably exceeding the sample sizes of previous network meta-analyses in this domain, permitted a formal leave-one-out sensitivity analysis across the complete dataset. The pooled odds ratio varied minimally (2.33–2.42) across all 30 iterations, with statistical significance preserved throughout, thereby providing a stronger empirical basis for confidence in the stability of the pooled estimate than has previously been available for this research question.

This study applied standardized risk-of-bias instruments—the Cochrane RoB 2 tool for randomized trials and ROBINS-I for observational studies—systematically across the entire body of included evidence, rather than to a limited subset. The majority of studies were judged to carry low risk of bias, with confounding in observational cohorts representing the most frequently identified source of moderate risk. When combined with structured funnel plot analysis and Egger’s regression testing, this approach also identified specific areas of publication bias—most notably within the iMRI and “other specialized techniques” subgroups—that earlier reviews, several of which lacked sufficient statistical power for meaningful bias assessment, were not positioned to detect.

These findings reaffirm the central conclusion of earlier, more narrowly focused analyses—that image-guided and neuronavigation-assisted resection improves surgical outcomes relative to conventional technique—while substantially broadening the evidentiary base, formalizing bias and sensitivity assessment, and establishing that this benefit is not confined to any single navigational technology. Accordingly, the principal contribution of this study to the literature is consolidative in nature: it reframes the central clinical question from whether a specific modality, such as iMRI or 5-ALA, is beneficial, toward the broader and more clinically meaningful inquiry of how consistently image-enhanced navigation, as a unifying surgical principle, improves outcomes across modalities. Nevertheless, the predominance of retrospective cohort designs, variability in outcome definitions, and detected publication bias within certain subgroups underscore the continued need for large, multicenter randomized trials employing harmonized long-term survival endpoints to confirm the durability of this benefit and to clarify whether any individual navigational modality confers superiority within specific glioma subtypes or anatomical locations.

## Conclusion

This meta-analysis of 30 studies spanning two decades demonstrates that neuronavigation-guided and image-enhanced glioma surgery is associated with significantly improved surgical outcomes compared with conventional resection, with a pooled odds ratio of 2.37 and low overall heterogeneity. The benefit was consistent across every technological subgroup examined—intraoperative MRI, ultrasound-based navigation, functional neuronavigation, advanced navigation technologies, and other specialized techniques—and remained stable under leave-one-out sensitivity analysis, indicating that the pooled effect is not driven by any single study or intervention category. Risk-of-bias assessment using RoB 2 and ROBINS-I indicated that the majority of included studies were at low risk of bias, lending further confidence to the overall findings, although publication bias was detected within the iMRI and other specialized technique subgroups and should be considered when interpreting the magnitude of benefit in these categories.

Taken together, these results support the continued adoption and integration of neuronavigation and advanced image-guided techniques into standard glioma surgical practice as a means of maximizing safe resection and improving patient outcomes. At the same time, the evidence base remains dominated by retrospective and single-center studies with heterogeneous outcome definitions, and long-term survival data are comparatively limited relative to resection-based endpoints. Large-scale, multicenter randomized controlled trials with standardized outcome reporting and extended follow-up are needed to confirm the durability of these benefits, to clarify the comparative efficacy of individual navigational modalities, and to determine which techniques offer the greatest advantage for specific glioma subtypes, grades, and anatomical locations.
